# *N*,*N*′-Bis(2,5-dichloro­phen­yl)isophthalamide

**DOI:** 10.1107/S1600536811031758

**Published:** 2011-08-11

**Authors:** Ruitao Zhu, Jinlong Dong, Yu Zuo, Yuehong Ren

**Affiliations:** a Department of Chemistry, Taiyuan Normal University, Taiyuan 030031, People’s Republic of China

## Abstract

The asymmetric unit of the title compound, C_20_H_12_Cl_4_N_2_O_2_, contains one half-mol­ecule with a center of symmetry along a C⋯C axis of the central benzene ring. The two C=O groups adopt an *anti* orientation and the two amide groups are twisted away from the central benzene ring by 27.38 (3) and 27.62 (4)°. The mean planes of the dichloro-substituted benzene rings are twisted by 7.95 (4)° with respect to the benzene ring. The crystal packing is stabilized by weak inter­molecular N—H⋯O inter­actions.

## Related literature

For the design of artificial receptors related to isophthalamide, see: Gale (2006 ▶). For related structures, see: Light *et al.* (2006 ▶); Kavallieratos *et al.* (1997 ▶, 1999 ▶). For standard bond lengths, see: Allen *et al.* (1987 ▶).
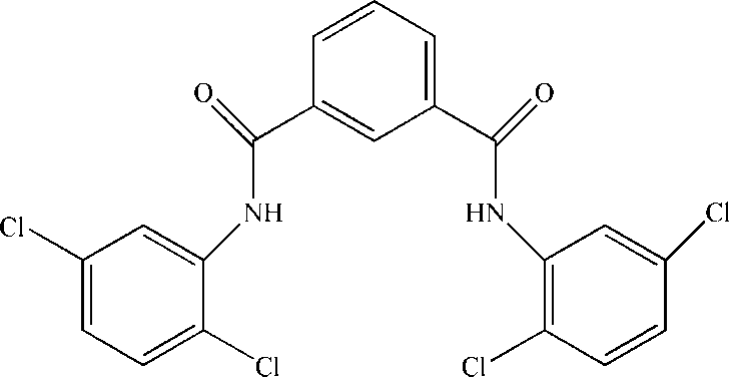


## Experimental

### 

#### Crystal data

C_20_H_12_Cl_4_N_2_O_2_
*M* *_r_* = 454.12
Monoclinic, 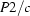

*a* = 11.3661 (11) Å
*b* = 10.0239 (9) Å
*c* = 8.9470 (7) Å
β = 109.988 (1)°
*V* = 957.95 (15) Å^3^
*Z* = 2
Mo *K*α radiation
μ = 0.64 mm^−1^
*T* = 298 K
0.49 × 0.20 × 0.10 mm

#### Data collection

Bruker SMART CCD area-detector diffractometer
Absorption correction: multi-scan (*SADABS*; Sheldrick, 2004 ▶) *T*_min_ = 0.745, *T*_max_ = 0.939
4668 measured reflections
1687 independent reflections
1152 reflections with *I* > 2σ(*I*)
*R*_int_ = 0.030

#### Refinement

*R*[*F* ^2^ > 2σ(*F* ^2^)] = 0.042
*wR*(*F* ^2^) = 0.110
*S* = 1.01
1687 reflections
128 parameters
H-atom parameters constrained
Δρ_max_ = 0.27 e Å^−3^
Δρ_min_ = −0.30 e Å^−3^

### 

Data collection: *SMART* (Bruker, 2007 ▶); cell refinement: *SAINT* (Bruker, 2007 ▶); data reduction: *SAINT*; program(s) used to solve structure: *SHELXS97* (Sheldrick, 2008 ▶); program(s) used to refine structure: *SHELXL97* (Sheldrick, 2008 ▶); molecular graphics: *SHELXTL* (Sheldrick, 2008 ▶) and *PLATON* (Spek, 2009 ▶); software used to prepare material for publication: *SHELXTL*.

## Supplementary Material

Crystal structure: contains datablock(s) I, global. DOI: 10.1107/S1600536811031758/jj2095sup1.cif

Structure factors: contains datablock(s) I. DOI: 10.1107/S1600536811031758/jj2095Isup2.hkl

Supplementary material file. DOI: 10.1107/S1600536811031758/jj2095Isup3.cml

Additional supplementary materials:  crystallographic information; 3D view; checkCIF report

## Figures and Tables

**Table 1:** Hydrogen-bond geometry (Å, °)

*D*—H⋯*A*	*D*—H	H⋯*A*	*D*⋯*A*	*D*—H⋯*A*
N1—H1⋯O1^i^	0.86	2.22	3.046 (3)	160

Symmetry code: (i) 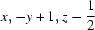.

**Fig. 1.**
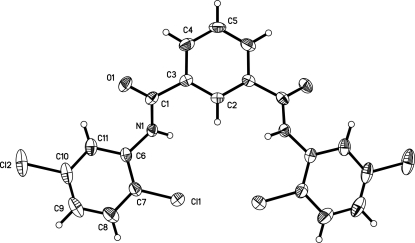
The molecular structure of the title compound with displacement ellipsoids are drawn at the 30% probability level.

## supplementary crystallographic information

### Comment

Anion recognition is an area of growing interest in supramolecular chemistry due to its important role in a wide range of environmental, clinical, chemical, and biological applications.  Considerable attention has been focused on the design of artificial receptors that are able to selectively recognize and sense anion species (Gale, 2006). Artificial receptors, containing the isophthalamide core function as effective receptors for halide anions in very simple systems (Kavallieratos *et al.*, 1997, 1999; Light *et al.* 2006). We report here the crystal structure of the title compound, C_20_H_12_Cl_4_N_2_O_2_, (I), related to these types of receptors.

In the title compound,(I), the asymmetric unit containing one-half of the molecule crystallizes with a center of symmetry along the C2—C5 axis in the benzene ring thereby producing the desired structure (Fig. 1).  In the molecule the two C═O groups adopt an *anti* orientation and the two amide groups are twisted away from the center benzene ring by 27.38 (3) ° and 27.62 (4) °, respectively.  The mean planes of the dichloro substituted benzene rings are twisted by 7.95 (4) ° with that of the benzene ring.  Bond lengths are in normal ranges (Allen *et al.*, 1987). Crystal packing is stabilized by weak N—H···O intermolecular interactions (Table 1).

### Experimental

*N,N*-Bis(2,5-dichlorophenyl)isophthalamide (I) was prepared according to literature procedures (Kavallieratos *et al.*, 1997, 1999). To dichloromethane (20 ml) in a 100 ml flask was added 2,5-dichloroaniline(1.62 g, 10 mmol) with magnetic stirring. Isophthalogyl chloride (1.01 g, 5 mmol) was added gradually, and the reaction mixture was stirred at room temperature for 2 h and then poured into ice water (100 ml). The product was precipitated as a white powder, which was washed three times with water. Recrystallization from dimethyl sulfoxide solution produced the crystals of the title compound.

### Refinement

H atoms were placed in idealized positions and allowed to ride on their respective parent atoms, with C—H = 0.93–0.96 Å, N—H = 0.86Å and *U*_iso_(H)= 1.2*U*_eq_(C,*N*) or 1.5*U*_eq_(C_methyl_).

### Crystal data

**Table:**

C_20_H_12_Cl_4_N_2_O_2_	*F*(000) = 460
*M**_r_* = 454.12	*D*_x_ = 1.574 Mg m^−^^3^
Monoclinic, *P*2/*c*	Mo *K*α radiation, λ = 0.71073 Å
Hall symbol: -P 2yc	Cell parameters from 1505 reflections
*a* = 11.3661 (11) Å	θ = 2.8–27.4°
*b* = 10.0239 (9) Å	µ = 0.64 mm^−^^1^
*c* = 8.9470 (7) Å	*T* = 298 K
β = 109.988 (1)°	Prism, colorless
*V* = 957.95 (15) Å^3^	0.49 × 0.20 × 0.10 mm
*Z* = 2	

### Data collection

**Table:**

Bruker SMART CCD area-detector diffractometer	1687 independent reflections
Radiation source: fine-focus sealed tube	1152 reflections with *I* > 2σ(*I*)
graphite	*R*_int_ = 0.030
φ and ω scans	θ_max_ = 25.0°, θ_min_ = 2.8°
Absorption correction: multi-scan (*SADABS*; Sheldrick, 2004)	*h* = −13→6
*T*_min_ = 0.745, *T*_max_ = 0.939	*k* = −11→11
4668 measured reflections	*l* = −10→10

### Refinement

**Table:**

Refinement on *F*^2^	Primary atom site location: structure-invariant direct methods
Least-squares matrix: full	Secondary atom site location: difference Fourier map
*R*[*F*^2^ > 2σ(*F*^2^)] = 0.042	Hydrogen site location: inferred from neighbouring sites
*wR*(*F*^2^) = 0.110	H-atom parameters constrained
*S* = 1.01	*w* = 1/[σ^2^(*F*_o_^2^) + (0.0416*P*)^2^ + 0.6846*P*] where *P* = (*F*_o_^2^ + 2*F*_c_^2^)/3
1687 reflections	(Δ/σ)_max_ < 0.001
128 parameters	Δρ_max_ = 0.27 e Å^−^^3^
0 restraints	Δρ_min_ = −0.30 e Å^−^^3^

### Special details

**Table:**

Geometry. All e.s.d.'s (except the e.s.d. in the dihedral angle between two l.s. planes) are estimated using the full covariance matrix. The cell e.s.d.'s are taken into account individually in the estimation of e.s.d.'s in distances, angles and torsion angles; correlations between e.s.d.'s in cell parameters are only used when they are defined by crystal symmetry. An approximate (isotropic) treatment of cell e.s.d.'s is used for estimating e.s.d.'s involving l.s. planes.
Refinement. Refinement of *F*^2^ against ALL reflections. The weighted *R*-factor *wR* and goodness of fit *S* are based on *F*^2^, conventional *R*-factors *R* are based on *F*, with *F* set to zero for negative *F*^2^. The threshold expression of *F*^2^ > σ(*F*^2^) is used only for calculating *R*-factors(gt) *etc*. and is not relevant to the choice of reflections for refinement. *R*-factors based on *F*^2^ are statistically about twice as large as those based on *F*, and *R*- factors based on ALL data will be even larger.

### Fractional atomic coordinates and isotropic or equivalent isotropic displacement parameters (Å^2^)

**Table:**

	*x*	*y*	*z*	*U*_iso_*/*U*_eq_	
Cl1	0.64968 (9)	0.85140 (8)	0.38003 (10)	0.0726 (3)	
Cl2	0.99324 (10)	0.70144 (15)	1.06068 (10)	0.1021 (5)	
N1	0.7062 (2)	0.5769 (2)	0.4952 (2)	0.0412 (6)	
H1	0.6911	0.5986	0.3974	0.049*	
O1	0.70204 (19)	0.4014 (2)	0.6539 (2)	0.0544 (6)	
C1	0.6692 (2)	0.4532 (3)	0.5224 (3)	0.0392 (7)	
C2	0.5000	0.4520 (4)	0.2500	0.0362 (9)	
H2	0.5000	0.5448	0.2500	0.043*	
C3	0.5811 (2)	0.3842 (3)	0.3794 (3)	0.0372 (6)	
C4	0.5787 (3)	0.2456 (3)	0.3786 (4)	0.0537 (8)	
H4	0.6306	0.1985	0.4657	0.064*	
C5	0.5000	0.1782 (4)	0.2500	0.0638 (13)	
H5	0.5000	0.0854	0.2500	0.077*	
C6	0.7666 (2)	0.6736 (3)	0.6099 (3)	0.0401 (7)	
C7	0.7463 (3)	0.8080 (3)	0.5689 (3)	0.0489 (8)	
C8	0.8009 (3)	0.9072 (4)	0.6768 (4)	0.0672 (10)	
H8	0.7873	0.9962	0.6468	0.081*	
C9	0.8753 (3)	0.8746 (4)	0.8289 (4)	0.0732 (11)	
H9	0.9104	0.9410	0.9034	0.088*	
C10	0.8969 (3)	0.7431 (4)	0.8689 (4)	0.0640 (10)	
C11	0.8451 (3)	0.6423 (3)	0.7628 (3)	0.0513 (8)	
H11	0.8624	0.5536	0.7932	0.062*	

### Atomic displacement parameters (Å^2^)

**Table:**

	*U*^11^	*U*^22^	*U*^33^	*U*^12^	*U*^13^	*U*^23^
Cl1	0.0832 (7)	0.0488 (5)	0.0643 (6)	0.0049 (4)	−0.0025 (5)	0.0043 (4)
Cl2	0.0748 (7)	0.1852 (13)	0.0349 (5)	−0.0308 (7)	0.0039 (4)	−0.0066 (6)
N1	0.0485 (14)	0.0452 (14)	0.0276 (11)	−0.0025 (11)	0.0101 (10)	0.0034 (10)
O1	0.0655 (14)	0.0594 (13)	0.0382 (11)	0.0103 (11)	0.0177 (10)	0.0161 (10)
C1	0.0390 (15)	0.0439 (16)	0.0397 (16)	0.0121 (13)	0.0197 (13)	0.0064 (13)
C2	0.041 (2)	0.0292 (19)	0.042 (2)	0.000	0.0197 (18)	0.000
C3	0.0366 (15)	0.0367 (15)	0.0427 (16)	0.0029 (12)	0.0191 (13)	0.0041 (12)
C4	0.0489 (18)	0.0418 (17)	0.069 (2)	0.0079 (15)	0.0184 (16)	0.0109 (15)
C5	0.069 (3)	0.027 (2)	0.089 (4)	0.000	0.019 (3)	0.000
C6	0.0347 (15)	0.0546 (18)	0.0327 (15)	−0.0042 (13)	0.0139 (12)	−0.0040 (13)
C7	0.0422 (17)	0.0531 (18)	0.0475 (17)	−0.0007 (14)	0.0101 (14)	−0.0103 (14)
C8	0.061 (2)	0.064 (2)	0.075 (2)	−0.0116 (18)	0.020 (2)	−0.0223 (19)
C9	0.056 (2)	0.102 (3)	0.061 (2)	−0.020 (2)	0.0185 (19)	−0.040 (2)
C10	0.0418 (18)	0.115 (3)	0.0355 (17)	−0.016 (2)	0.0131 (14)	−0.0138 (19)
C11	0.0395 (16)	0.077 (2)	0.0369 (16)	−0.0049 (16)	0.0128 (14)	0.0011 (16)

### Geometric parameters (Å, °)

**Table:**

Cl1—C7	1.727 (3)	C4—H4	0.9300
Cl2—C10	1.742 (3)	C5—C4^i^	1.370 (4)
N1—C1	1.358 (3)	C5—H5	0.9300
N1—C6	1.408 (3)	C6—C11	1.390 (4)
N1—H1	0.8600	C6—C7	1.395 (4)
O1—C1	1.222 (3)	C7—C8	1.376 (4)
C1—C3	1.498 (4)	C8—C9	1.373 (5)
C2—C3^i^	1.387 (3)	C8—H8	0.9300
C2—C3	1.387 (3)	C9—C10	1.366 (5)
C2—H2	0.9300	C9—H9	0.9300
C3—C4	1.389 (4)	C10—C11	1.374 (5)
C4—C5	1.370 (4)	C11—H11	0.9300
			
C1—N1—C6	127.0 (2)	C11—C6—C7	118.0 (3)
C1—N1—H1	116.5	C11—C6—N1	123.5 (3)
C6—N1—H1	116.5	C7—C6—N1	118.5 (2)
O1—C1—N1	123.3 (3)	C8—C7—C6	121.2 (3)
O1—C1—C3	121.4 (3)	C8—C7—Cl1	119.2 (3)
N1—C1—C3	115.2 (2)	C6—C7—Cl1	119.6 (2)
C3^i^—C2—C3	121.2 (3)	C9—C8—C7	120.0 (3)
C3^i^—C2—H2	119.4	C9—C8—H8	120.0
C3—C2—H2	119.4	C7—C8—H8	120.0
C2—C3—C4	118.7 (3)	C10—C9—C8	118.9 (3)
C2—C3—C1	123.1 (2)	C10—C9—H9	120.5
C4—C3—C1	118.2 (2)	C8—C9—H9	120.5
C5—C4—C3	120.2 (3)	C9—C10—C11	122.2 (3)
C5—C4—H4	119.9	C9—C10—Cl2	119.1 (3)
C3—C4—H4	119.9	C11—C10—Cl2	118.7 (3)
C4—C5—C4^i^	120.9 (4)	C10—C11—C6	119.5 (3)
C4—C5—H5	119.6	C10—C11—H11	120.2
C4^i^—C5—H5	119.6	C6—C11—H11	120.2
			
C6—N1—C1—O1	−12.3 (4)	C11—C6—C7—C8	−0.8 (5)
C6—N1—C1—C3	166.2 (2)	N1—C6—C7—C8	178.8 (3)
C3^i^—C2—C3—C4	−0.9 (2)	C11—C6—C7—Cl1	179.9 (2)
C3^i^—C2—C3—C1	−179.2 (3)	N1—C6—C7—Cl1	−0.6 (4)
O1—C1—C3—C2	151.0 (2)	C6—C7—C8—C9	−1.0 (5)
N1—C1—C3—C2	−27.5 (3)	Cl1—C7—C8—C9	178.3 (3)
O1—C1—C3—C4	−27.4 (4)	C7—C8—C9—C10	2.0 (5)
N1—C1—C3—C4	154.2 (3)	C8—C9—C10—C11	−1.1 (6)
C2—C3—C4—C5	1.9 (4)	C8—C9—C10—Cl2	179.0 (3)
C1—C3—C4—C5	−179.7 (2)	C9—C10—C11—C6	−0.8 (5)
C3—C4—C5—C4^i^	−1.0 (2)	Cl2—C10—C11—C6	179.1 (2)
C1—N1—C6—C11	29.4 (4)	C7—C6—C11—C10	1.7 (4)
C1—N1—C6—C7	−150.1 (3)	N1—C6—C11—C10	−177.8 (3)

Symmetry codes: (i) −*x*+1, *y*, −*z*+1/2.

### Hydrogen-bond geometry (Å, °)

**Table:**

*D*—H···*A*	*D*—H	H···*A*	*D*···*A*	*D*—H···*A*
N1—H1···O1^ii^	0.86	2.22	3.046 (3)	160.

Symmetry codes: (ii) *x*, −*y*+1, *z*−1/2.
